# Green Synthesis of AuNPs by *Acinetobacter* sp. GWRVA25: Optimization, Characterization, and Its Antioxidant Activity

**DOI:** 10.3389/fchem.2020.00474

**Published:** 2020-06-18

**Authors:** Shradhda B. Nadhe, Sweety A. Wadhwani, Richa Singh, Balu A. Chopade

**Affiliations:** ^1^Department of Microbiology, Savitribai Phule Pune University, Pune, India; ^2^Department of Biotechnology, SIES College of Arts, Science and Commerce (Autonomous), Mumbai, India; ^3^Dr. Babasaheb Ambedkar Marathwada University, Aurangabad, India

**Keywords:** *Acinetobacter* sp., gold nanoparticles, physicochemical optimization, characterization, spherical, antioxidant

## Abstract

Bacteriogenic synthesis of metal nanoparticles is ecofriendly and greatly influenced by physico-chemical reaction parameters with respect to shape and size. Thus, present work aimed to synthesize and optimization of bacteriogenic gold nanoparticles (AuNPs) and study their antioxidant activity. *Acinetobacter* sp. cells were able to synthesize AuNPs, when challenged with tetra-chloroauric acid (HAuCl_4_). By physicochemical optimization, maximum synthesis was obtained with 72 h old culture using 2.1 × 10^9^ CFU/ml cell density. Whereas, pH-7 is suitable for AuNPs synthesis. HAuCl_4_ concentration (0.5 mM) enhanced the formation of monodispersed and spherical nanoparticles (15 ± 10 nm). At 37°C temperature, *Acinetobacter* sp. released nanoparticles in supernatant. From characterization, AuNPs were found to be crystalline in nature with negative surface charge. AuNPs showed up to 86% different radical scavenging ability, exhibiting antioxidant activity. In conclusion, spherical AuNPs can be synthesized using *Acinetobacter* sp. through physicochemical optimization. This is the first report of antioxidant activity exhibited by monodispersed bacteriogenic AuNPs synthesized using *Acinetobacter* sp.

## Introduction

Biological methods of synthesis of nanoparticles are ecofriendly with easy scale up processes (Makarov et al., 2014). Various biological systems such as plants, fungi, bacteria, biomolecules, etc. are reported to synthesize nanoparticles (Ghosh et al., 2012; Singh et al., 2013; Shedbalkar et al., 2014; Wadhwani et al., 2014, 2018; Yuan et al., 2017; Molnár et al., 2018; Onitsuka et al., 2019). Bacterial system is beneficial over others in production of customized nanoparticles by controlling physico-chemical parameters. *Acinetobacter* is ubiquitous in nature with high survival rate (Towner and Chopade, 1987; Shakibaie et al., 1999; Sahu et al., 2012; Fulsundar et al., 2014, 2015; Wong et al., 2017). It is found in diverse environments such as rhizosphere soil, hospitals, sewage water, on human or animal skin, food, etc. (Patil et al., 2001; Saha and Chopade, 2002; Yavankar et al., 2007; Chopade et al., 2008; Jagtap et al., 2009; Sachdev et al., 2010; Farokh et al., 2011; Pour et al., 2011; Yele et al., 2012; Mujumdar et al., 2014; Wadhwani et al., 2014; Jagtap and Chopade, 2015). It can withstand extreme conditions such as high antibiotics, radiations, desiccation, and metal salts (Deshpande and Chopade, 1994; Dhakephalkar and Chopade, 1994; Shakibaie et al., 1998; Fulsundar et al., 2014, 2015). *Acinetobacter* are reported to synthesize various metal nanoparticles viz. silver, platinum and gold (Gaidhani et al., 2013, 2014; Singh et al., 2013, 2015, 2018; Wadhwani et al., 2014). *Acinetobacter* spp. are isolated from sewage have ability to synthesize polyhedral gold nanoparticles on optimization of physiochemical parameters (Wadhwani et al., 2014). In view of this *Acinetobacter* group of microorganisms is excellent for the synthesis of AuNPs. In order to get monodisperse nanoparticles using bacterial system, one has to optimize its synthesis by controlling physiochemical environment (Gaidhani et al., 2013; Wadhwani et al., 2014; Singh et al., 2015; Nadhe et al., 2019). It is possible to get nanoparticles of desired shape and size by maintaining culture age, cell density, pH, metal salt concentration and temperature (Wadhwani et al., 2014).

Applications of nanoparticles are highly dependent on its properties which is controlled by size and shape (Huang and El-Sayed, 2010; Ghosh et al., 2015a; Ghosh and Jini Chacko, 2016). AuNPs has tremendous applications in medicine, such as treatment of diabetes mellitus, cancer, cardiovascular diseases, and in control of multiple antibiotic resistance in human pathogens as a novel nano-antibiotics against tuberculosis (Giljohann et al., 2010; Kitture et al., 2012; Ghosh et al., 2014, 2015c; Mallick et al., 2015; Singh et al., 2016; Wadhwani et al., 2017). Antioxidant properties of nanoparticles is one of the most important applications in therapeutics and biomedical field (Kitture et al., 2014; Salunke et al., 2014; Ghosh et al., 2015b; Ghosh and Jini Chacko, 2016). Reactive oxygen species (ROS) are generated as by product of cellular metabolism resulting in oxidative stress. This stress cause for damage of DNA, proteins and lipids, eventually leading cellular aging and death. Moreover, ROS levels inside the cell also hampers cell signaling process responsible for imped cell proliferation, which is the most imperative factor of cancer (Schieber and Chandel, 2014). Consumption of antioxidants as dietary supplements has helped people to fight against disorders like cancer, cardiovascular diseases and aging (Watters et al., 2007; Bjelakovic et al., 2014). AuNPs synthesized by chemical, physical, and biological routes possess antioxidant activity (Yakimovich et al., 2008; Medhe et al., 2014; Madhanraj et al., 2017). There are very few reports on antioxidant properties of AuNPs synthesized by microorganisms (Ahmad et al., 2003; Veeraapandian et al., 2012; Manivasagan et al., 2015; Markus et al., 2016). However, there is no report on antioxidant properties of AuNPs synthesized by *Acinetobacter* sp. Therefore, we proposed a hypothesis that physicochemical parameters optimization may affect the morphology of AuNPs synthesized using *Acinetobacter* sp. isolated from wheat rhizosphere and help in getting monodispersed desired shape and size nanoparticles. Also, optimized AuNPs may exhibit antioxidant activity.

## Materials and Methodology

### Chemicals

HAuCl_4_, Sodium hydroxide (NaOH) and hydrochloric acid (HCl) were purchased from SRL, Mumbai, India. Luria Bertani (LB) broth, 2,2-diphenyl-2-picrylhydrazyl (DPPH), sodium nitroprusside, hydrogen peroxide (H_2_O_2_) sulphanilamide and naphthylamine were procured from HiMedia, Mumbai, India. One to ten, phenanthroline was purchased from Merck, Mumbai, India.

### Cultures Used and Growth Condition

Fourteen cultures of *Acinetobacter* spp. isolated from wheat rhizosphere and identified by 16S rRNA sequencing (NCBI accession number- EU921457, EU921459-EU921464, EU921468, EU921470-EU921472, EU221350, EU221386, EU221389), were selected for screening (Sachdev et al., 2010). All cultures were grown overnight in LB broth at 30°C, 150 rpm and preserved as glycerol stocks at −80°C.

### Cultures Screening for AuNPs Synthesis

For preliminary screening, 18 h grown cultures (10 ml) were centrifuged at 8,000 rpm for 10 min at 4°C. Cell pellet was collected and washed thrice and finally suspended in sterile milli-Q water. HAuCl_4_ was added to the suspension at 1 mM final concentration and incubated at 30°C. The suspension was checked for synthesis of AuNPs at regular interval by recording its UV-visible spectrum between 200 and 800 nm. Cell suspension and 1 mM HAuCl_4_ solution were kept as controls. The culture showing highest characteristic peak (between 500 to 560 nm) for presence of AuNPs was selected for further experiments.

### Optimization of AuNPs Synthesis

In order to get monodispersed nanoparticles physiochemical parameters were optimized by single factor optimization method. First of all, the culture age was optimized by challenging 6, 12, 18, 24, 48, 72, and 96 h grown cultures with 1 mM HAuCl_4_ salt at 30°C. Cell density of optimized age culture was adjusted to <0.3, 0.3, 0.6, 0.9, 1.2, 1.5, 1.8, 2.1, 2.4, 2.7, and 3 × 10^9^ CFU/ml as per McFarland's standards and allowed to react at 30°C with 1 mM HAuCl_4_. To check effect of salt concentration on AuNPs synthesis OD adjusted culture was combined with different HAuCl_4_ concentrations from 0.1 to 4 mM. Moreover, pH of the reaction mixture was adjusted with the help of 0.1 N HCl and NaOH ranging from 2 to 10 to obtained optimized pH value and then challenged with optimized HAuCl_4_ concentrations. Further, optimization of temperature was carried out by incubating the reaction mixture at different temperatures such as 8, 20, 30, 37, 50, 60°C. UV- visible spectra (200–800 nm) were used to monitor the reaction for maximum and monodispersed synthesis of AuNPs. Moreover, transmission electron microscope (TEM) Technai G2, 20 ultra-win FEI, Netherlands, was used to monitor shape and size of the AuNPs to get monodispersed particles.

### Characterization of AuNPs

Depending on location of nanoparticles, course of action was decided for separation of nanoparticles. Nanoparticles were separated from cell pellet by centrifugation at 8,000 rpm for 7 min. The separated cell pellet was re-suspended in equal volume of milli-Q water and subjected to sonication at 25% amplitude for 30 on and 10 s off cycle on ice for 30 min. Again cell debris were separated by centrifugation at 8,000 rpm for 7 min. from the supernatant the nanoparticles were concentrated using centrifugation at 12,000 rpm at 4°C for 20 min. Further nanoparticles washed twice with sterile milli-Q water and collected by centrifugation at 12,000 rpm at 4°C for 20 min. The nanoparticles pellet was reconstituted in 5 ml of milli-Q water.

The nanoparticles were characterized using several techniques. A thin film of AuNPs was coated on glass slide and analyzed for X-ray diffraction pattern using D8 Advanced Brucker X-ray diffractometer. For scanning electron microscopy, a drop was layered on 0.5 × 0.5 cm glass piece and air dried at room temperature (RT). The glass piece was coated with platinum with the help of sputter coater and analyzed under field emission scanning electron microscope (FESEM), FEI Nova NanoSEM 450. The size, shape and fringes pattern of AuNPs was determined using TEM, Technai G2, 20 ultra-win FEI, Netherlands. A drop of nanoparticles coated on copper grid was used for TEM analysis. The elemental content of nanoparticles was determined by energy dispersive spectroscopy (EDS) with energy dispersive X-ray spectrophotometer (JED-2300; JEOL) assisted with TEM. Also, selected area electron diffraction (SAED) was studied using same grid. Cell pellet used for synthesis of AuNPs and synthesized AuNPs was air dried and FTIR spectrum was recorded from 380 to 4,000 cm^−1^ at resolution of 2 cm^−1^ using Bruker tensor 37 FTIR spectrophotometer. A diluted solution of AuNPs subjected to analysis of particle size distribution and zeta potential using dynamic light scattering (DLS) technology with the help of zetasizer (Nano-ZS90, Malvern, UK).

### Antioxidant Activity of AuNPs

#### DPPH Scavenging Assay

Synthesized nanoparticles were separated from cells by sonication and centrifuged at 6,000 rpm for 5 min to get rid of cell residues. Antioxidant activity of AuNPs (25–150 μg) studied by free radicals scavenging ability from DPPH (Medhe et al., 2014). Different concentrations of AuNPs mixed with ethanolic DPPH and incubated for 30 min in dark. The developed product was read spectrophotometrically at 517 nm. Only water kept as blank. Percent scavenging activity was calculated by following formula:

$$\begin{array}{l} \text{Percent scavenging activity }\left(\text{\%}\right)=\frac{{\text{A}}_{\text{Blank}}-\;{\text{A}}_{\text{Sample}}}{{\text{A}}_{\text{Blank}}}\times 100 \end{array}$$

Where, A_Blank_ = absorbance of blank

A_Sample_ = absorbance of sample

The experiment was performed in triplicate and value is expressed as mean ± s.d.

#### Nitric Oxide (NO^−^) Scavenging Assay

For nitric oxide scavenging assay 1 ml of different concentrations of AuNPs (25–150 μg/ml) was first mixed with 0.5 ml sodium nitroprusside (1 mM) prepared in phosphate buffer saline (PBS). The mixture was incubated at RT for 180 min, followed by addition of equal amount Griess reagent (1% sulphanilamide and 0.1% naphthylamine prepared separately in 2.5% phosphoric acid and mixed together). The colored product developed was read at 542 nm spectrophotometrically. Only PBS buffer was kept as control and ascorbic acid kept as standard (Boora et al., 2014). Percent NO^−^ scavenging activity of was calculated by formula mentioned above. The experiment was performed in triplicate and value is expressed as mean ± s.d.

#### H_2_O_2_ Scavenging Assay

In this assay different concentrations of 1.5 ml AuNPs (25–150 μg/ml) was first mixed with 0.25 ml ammonium ferrous sulfate (1 mM). To this mixture, 0.0625 ml of 5 mM H_2_O_2_ solution was added and incubated for 5 min in dark at RT. After incubation 1 mM 1,10-phenanthrolin was added to each tube and incubated at RT for 10 min. All tubes were read at 510 nm using spectrophotometer. The blank solution contained only water, ferrous ammonium sulfate and 1, 10- phenanthroline. Ascorbic acid kept as antioxidant standard (Mukhopadhyay et al., 2016). Percent H_2_O_2_ scavenging activity was calculated by formula mentioned above. The experiment was performed in triplicate and value is expressed as mean ± s.d.

## Results and Discussion

### Screening for AuNPs Synthesis

**Figure 1:**
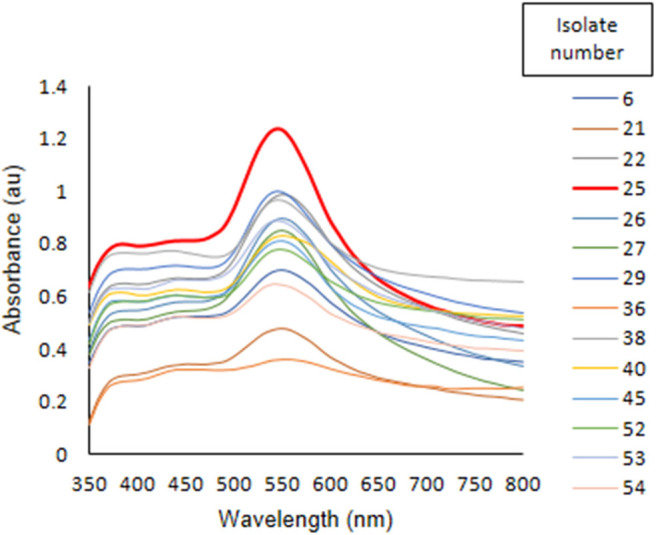
Screening of 14 isolates of *Acinetobacter* spp. for synthesis of AuNPs.

All isolates of *Acinetobacter* spp. screened for synthesis of AuNPs, showed positive result when cell suspension was challenged with 1 mM HAuCl_4_ (Figure 1). *Acinetobacter* spp. isolated from rhizosphere are known to synthesize silver and platinum nanoparticles (Gaidhani et al., 2013; Singh et al., 2013). However, this is the first report on synthesis of AuNPs using biomass *Acinetobacter* spp., having plant growth promoting properties, isolated from wheat rhizosphere soil (Sachdev et al., 2010). There is a report available on synthesis of AuNPs using *Acinetobacter* sp. SW30 which was isolated from activated sewage sludge (Wadhwani et al., 2014). Later on, Wadhwani et al. (2018) suggested that an enzyme lignin peroxidase present in *Acinetobacter* sp. SW30, is involved in reduction of HAuCl_4_ to spherical AuNPs. In UV-visible spectrum analysis, a surface plasmon resonance (SPR) peak was observed at 550 nm which is characteristic peak for AuNPs synthesis (Figure 1). *Acinetobacter* sp. GWRVA25 gave maximum synthesis of AuNPs with highest peak at 550 nm with color change to purple. SPR is shown by AuNPs due to oscillation of free electrons on incidence by light of particular wavelength. In case of AuNPs, color change is observed from yellow to red and in some cases mauve to purple, depending upon size, and shape of nanoparticles. Green color was also observed in case of rod-shaped nanoparticles (Murphy et al., 2008). The varying color change of nanoparticles is due the absorption and emission of particular wavelength which lies in visible region of electromagnetic spectrum (Murphy et al., 2008; Zhang et al., 2016). Culture of *Acinetobacter* sp. GWRVA25 (NCBI accession number EU921461) was deposited at Microbial Culture Collection (MCC), National Center for Cell Sciences, Pune, India. MCC accession number for culture was MCC 3368.

### Optimization of AuNPs Synthesis

**Figure 2:**
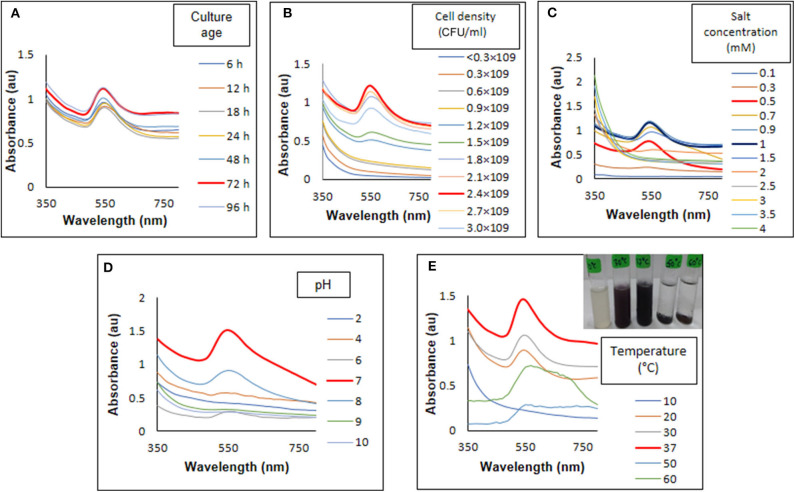
Optimization of physicochemical parameter for synthesis of AuNPs. **(A)** Culture age, **(B)** cell density, **(C)** salt concentration, **(D)** pH, and **(E)** temperature.

In case of bacteriogenic nanoparticles synthesis, shape, and size of nanoparticles can be controlled by optimization of physicochemical parameters (Singh et al., 2013, 2015; Wadhwani et al., 2014). It is an advantageous process over other biogenic and chemical or physical synthesis of nanoparticles. During optimization of culture age, maximum synthesis of AuNPs was observed in both 72 and 96 h grown cultures with 1 mM HAuCl_4_ (Figure 2A). When cell density of 72 h grown culture was adjusted from 0.3 × 10^9^ to 3 × 10^9^ CFU/ml, highest synthesis observed at 2.4 × 10^9^ CFU/ml (Figure 2B). This is may be due to the number of biomolecules required for synthesis of AuNPs present in 2.4 × 10^9^ CFU/ml density. Moreover, biomolecules required for the synthesis of AuNPs may occurred in late stationary phase at their highest. During synthesis of nanoparticles using *Acinetobacter* sp. culture age vary as the strain varies. In contrast to our result, for the synthesis AuNPs using *Acinetobacter* sp. SW30 the optimum age is 24 h, a late log phase culture (Wadhwani et al., 2014).

**Figure 3:**
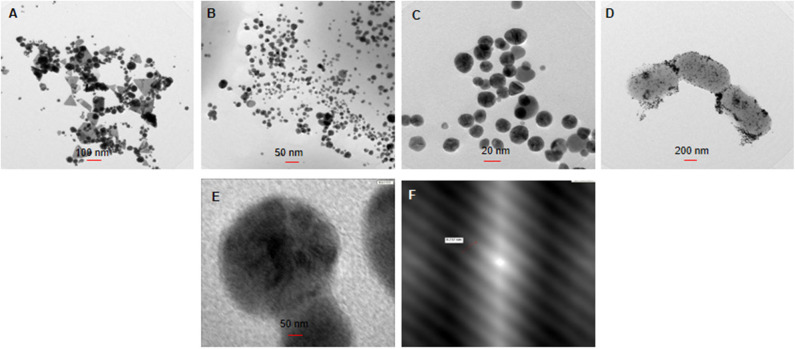
Transmission electron microscopy for the synthesis of AuNPs, using **(A,B)** 1 and 0.5 mM of HAuCl_4_, respectively, **(C,D)** at 37 and 50°C, **(E)** enlarged image of optimized AuNPs, and **(F)** lattice and fringes on AuNP.

Further, HAuCl_4_ concentration shown to have effect on shape and size of nanoparticles (He et al., 2008). From UV-visible spectrum analysis, highest synthesis was observed with 1 mM HAuCl_4_ (Figure 2C). However, when these particles visualized under TEM (Figure 3A), particles were found to be poly-dispersed with different shapes such as triangles spheres and polyhedron. As compared to 1 mM, particles formed at 0.5 mM salt concentration were more monodispersed, spherical with size 15 ± 10 nm (Figure 3B). besides this there was a blue shift in UV-visible spectrum from 550 to 540 nm indicating formation of smaller particles at 0.5 mM HAuCl_4_ concentration (Figure 2C). *Rhodopseudomonas capsulate* has been reports to synthesize small and uniform spherical shaped nanoparticles at low concentrations of HAuCl_4_ (0.25 mM). As the concentration increased (0.3–0.5 mM), size of the nanoparticles increased and formation of nanowires observed as intermediate product. Relative concentration of reducing proteins to the gold ions affect the thermodynamic control in both nucleation and growth of nanoparticles formation (He et al., 2008). Similar observations were noted by Wadhwani et al. (2014) in case of AuNPs synthesized using *Acinetobacter* sp. SW30.

pH of the reaction influences the shape of the particles very well. In our study, pH 7 was found to be the most suitable for synthesis for AuNPs (Figure 2D). Another study by Wadhwani et al. (2014) showed the formation of polyhedral 20 ± 10 nm sized nanoparticles at pH-9. In contrast, synthesis of AuNPs using *Verticillium luteoalbum* is favored in acidic pH which are intracellular (Gericke and Pinches, 2006). It has also been observed that alkaline pH favors the extracellular synthesis of the nanoparticles which are polyhedral in shape (Wadhwani et al., 2014). The reaction was further optimized by incubating the reaction mixture at different temperatures. UV-visible spectrum (Figure 2E) analysis showed that 37°C was most suitable temperature for AuNPs synthesis. Temperature of the reaction is one of the important factors in nanogold synthesis because it affects activity of biomolecules. Majority of the reports demonstrated enhanced effect of the synthesis of nanoparticles with high temperature (Gericke and Pinches, 2006; Mohammed Fayaz et al., 2009). However, in our case, 37°C was the ambient temperature. At high temperature cells produced intracellular nanoparticles which settle at the bottom, thereby, making it difficult to separate nanoparticles from the cells (Figure 2E). Moreover, TEM analysis of AuNPs synthesized at 37°C (Figure 3C) showed monodispersed nanoparticles whereas at 50°C (Figure 3D) nanoparticles were attached to *Acinetobacter* cells.

### Characterization of AuNPs

**Figure 4:**
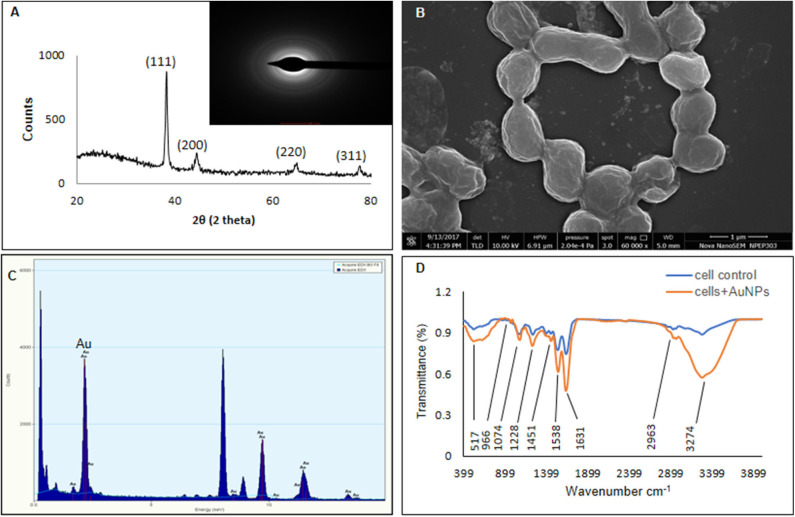
Characterization of AuNPs **(A)** XRD, **(B)** FESEM, **(C)** EDS, and **(D)** FTIR.

Crystalline nature of AuNPs was confirmed with XRD data when compared with standards (JCPDS file no: 04-0783). XRD pattern showed four peaks 38.10°, 44.1°, 64.5°, and 77.6° at 2θ corresponding to 111, 200, 220, and 311 plane indicating formation of crystalline AuNPs (Figure 4A). SAED pattern of AuNPs displayed four rings of reflection which arise from 111, 200, 220, and 311 lattice planes of face centered cubic crystalline structure (Figure 4A). The XRD and SAED pattern was similar to AuNPs synthesized using *Klebsiella pneumoniae* and *Acinetobacter* sp. SW30 (Wadhwani et al., 2014; Rajeshkumar, 2016). FESEM (Figure 4B) and TEM (Figure 3C), both revealed the formation of spherical cell bound and cell free AuNPs which was further confirmed to be of gold by EDS analysis indicating a peak at 2 keV (Figure 4C). FESEM revealed some degree of aggregation among nanoparticles. TEM showed the presence of spherical nanoparticles with average size of 15 ± 5 nm. The magnified image (Figure 3E) of nanoparticles taken under TEM showed lattice fringes structure with 0.23 nm distance between two lattices (Figure 3F). In earlier reports, TEM helped in determining the location of nanoparticles that is whether they are cell bound or intracellular or free in medium (Shedbalkar et al., 2014; Wadhwani et al., 2014; Singh et al., 2015). Nanoparticles can attach to cells with stability via cell bound proteins which tend to hold them (Shedbalkar et al., 2014). Loosely bound or intracellular nanoparticles can be separated by application of external force such as sonication, repetitive freeze thawing or any other cell bursting techniques (Wadhwani et al., 2014). Based on location of nanoparticles, course of action is decided for separation of nanoparticles. In our case, all three types of nanoparticles viz. cell free, cell bound and intracellular were found. Cell free particles were separated by only centrifugation processes while to get intracellular and cell bound nanoparticles, cell pellet was subjected to sonication followed by centrifugation at 12,000 rpm at 4°C.

In FTIR analysis (Figure 4D), FTIR spectrum of cells involved in AuNPs synthesis showed peak at 1,632 cm^−1^ for presence of amide II group. Peak at 3,276 cm^−1^ represents existence of O-H stretching which is for alcohols and phenols. After addition of HAuCl_4_, a new peak formed at 524 cm^−1^ indicates chloride formation and C-Cl stretching. FTIR of formed AuNPs significant increase in peak at 3,274, 1,631, and 1,538 cm^−1^ which showed presence of 0-H stretching, amide and nitro groups, respectively. Such enhancement of band of at 1,632, 1,535, and 1,232 cm^−1^ which are the bands for amide I-III indicates presence of proteins/polypeptides on the surface of nanoparticles. These molecules coat the nanoparticles rendering stabilization (Kalishwaralal et al., 2010). Many bacteria such as *Brevibacterium casei, Geobacillus stearothermophilus, Shewanella oneidensis, Acinetobacter* sp. SW30 showed involvement of amide group I–III bioreduction of HAuCl_4_ to AuNPs (Kalishwaralal et al., 2010; Mohammed Fayaz et al., 2011; Suresh et al., 2011; Wadhwani et al., 2016).

**Figure 5:**
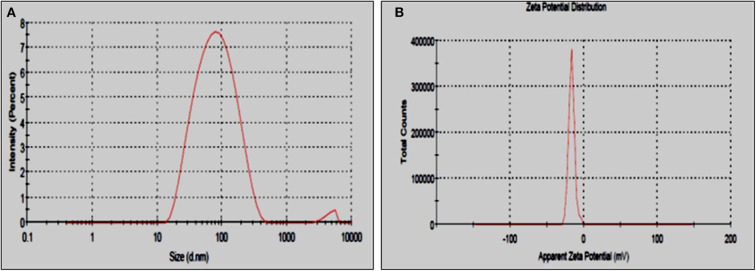
**(A)** DLS and **(B)** zeta potential of optimized AuNPs.

DLS analysis of optimized AuNPs showed presence of maximum 60 nm sized nanoparticles (Figure 5A). This size is far greater than the size observed using TEM. This difference can be explained by the fact that DLS is more partial toward detecting the larger sized particles and that it also considers hydrodynamic diameter of the particles (Jang et al., 2015). Zeta potential confirms the presence of negative (−17.4 mV) charge on the surface of AuNPs (Figure 5B). The negative charge prevents the agglomeration of nanoparticles by the negative- negative charge repulsion. Presence of charge on the nanoparticles is dependent on condition of the synthesis process and bacterium used (Li et al., 2016; Składanowski et al., 2017).

### Antioxidant Activity of AuNPs

#### DPPH Radical Scavenging Assay

**Figure 6:**
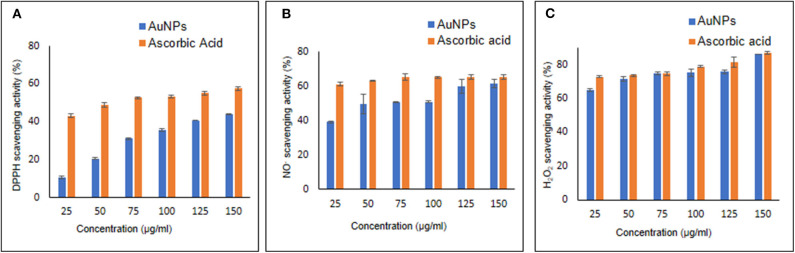
Antioxidant activity estimated by **(A)** DPPH scavenging activity, **(B)** NO^−^ scavenging assay, and **(C)** H_2_O_2_ scavenging assay.

DPPH is a free radical which changes its color from violet to yellow on reduction by a hydrogen or electron (Medhe et al., 2014). In DPPH scavenging assay, the compounds which are able to reduced DPPH are considered as antioxidants. By DPPH radical scavenging assay, it was found that AuNPs were able to react with free oxygen radicals and hence, possessed antioxidant activity. Figure 6A represent percent DPPH scavenging activity of increasing concentrations of AuNPs (sphere shaped). Up to 45% radical scavenging activity was seen with 150 μg/ml AuNPs. Whereas, AuNPs (polyhedral in shape) synthesized using *Acinetobacter* SW30 isolate did not show antioxidant properties (Wadhwani, 2017). The biological activity of nanoparticles is depending upon the aspect ratio of particles. Nanoparticles with high aspect ratio has been demonstrated to exhibit good antioxidant and antimicrobial properties (Sharma et al., 2015).

#### Nitric Oxide Scavenging Assay

Due to presence of a free unpaired electron, NO^−^ is considered as free radical that can interact with proteins and other free radicals. A highly reactive anion, peroxynitrite (ONOO^−^), is formed when NO^−^ and peroxide radical come together (Nagmoti et al., 2012). In nitric oxide scavenging assay, sodium nitroprusside reacts with oxygen to form nitrite. The nitrite ions diazotize with sulphanilamide. Further diazotized nitrate ions coupled with naphthyl ethylene diamine which results in formation of a pink colored complex. On the other hand, antioxidant donate a proton to nitrite ion, making it unavailable to react and thus decrease in absorbance was observed (Boora et al., 2014). Percent NO^−^ scavenging activity of AuNPs and ascorbic acid has been shown in Figure 6B NO^−^ scavenging activity of AuNPs increased with the concentration with maximum at 150 μg/ml. Around 61% NO^−^ scavenging activity was observed at 150 μg/ml concentration of AuNPs while standard ascorbic acid showed 65% activity with same concentration indicating comparable activity of our AuNPs.

#### H_2_O_2_ Scavenging Assay

1,10- phenanthroline assay is highly specific, reproducible, and rapid spectrophotometric method for detection of H_2_O_2_ scavenging activity *in vitro* environment (Mukhopadhyay et al., 2016). An anti-oxidant compound donates electron to H_2_O_2_ ions and thus neutralize it to water (Medhe et al., 2014). Synthesized AuNPs were able to scavenged 87% using 150 μg/ml concentration (Figure 6C). He et al. (2012) reported that AuNPs can catalyze rapid decomposition of H_2_O_2_. H_2_O_2_ scavenging activity shown by AuNPs is almost equal to other embedded 3,6-dihydroxyflavone AuNPs, used to enhance the antioxidant activity (Medhe et al., 2014).

## Conclusions

In conclusion, this study has successfully shown that *Acinetobacter* sp. GWRVA25 is able synthesize spherical, monodispersed crystalline AuNPs at neutral pH. Physicochemical parameters such as HAuCl_4_ concentration, temperature, and pH are the major factors that affect the shape and size of the bacteriogenic synthesis of AuNPs. Moreover, optimized AuNPs possess antioxidant activity. This is the first report of antioxidant activity shown by AuNPs synthesized using *Acinetobacter* sp. It would be interesting to decode molecular mechanism involved in antioxidant activity of AuNPs.

## Data Availability Statement

The raw data supporting the conclusions of this article will be made available by the authors, without undue reservation.

## Author Contributions

SN: conceptualization, methodology, investigation, validation, and writing—original draft. SW: conceptualization, methodology, investigation, validation, and editing. RS: investigation, writing—original draft, and editing. BC: conceptualization, writing—review and editing, supervision, project administration, resources, and funding acquisition.

## Conflict of Interest

The authors declare that the research was conducted in the absence of any commercial or financial relationships that could be construed as a potential conflict of interest.
